# Optical control of protein phosphatase function

**DOI:** 10.1038/s41467-019-12260-z

**Published:** 2019-09-26

**Authors:** Taylor M. Courtney, Alexander Deiters

**Affiliations:** Department of Chemistry, University of Pittsburgh, Pittsburgh, PA 15260 USA

**Keywords:** Optogenetics, Phosphorylation, Proteins, Phosphoproteins, Synthetic biology

## Abstract

Protein phosphatases are involved in embryonic development, metabolic homeostasis, stress response, cell cycle transitions, and many other essential biological mechanisms. Unlike kinases, protein phosphatases remain understudied and less characterized. Traditional genetic and biochemical methods have contributed significantly to our understanding; however, these methodologies lack precise and acute spatiotemporal control. Here, we report the development of a light-activated protein phosphatase, the dual specificity phosphatase 6 (DUSP6 or MKP3). Through genetic code expansion, MKP3 is placed under optical control via two different approaches: (i) incorporation of a caged cysteine into the active site for controlling catalytic activity and (ii) incorporation of a caged lysine into the kinase interaction motif for controlling the protein-protein interaction between the phosphatase and its substrate. Both strategies are expected to be applicable to the engineering of a wide range of light-activated phosphatases. Applying the optogenetically controlled MKP3 in conjunction with live cell reporters, we discover that ERK nuclear translocation is regulated in a graded manner in response to increasing MKP3 activity.

## Abstract (web summary)

Protein phosphatases play an essential role in signal transduction, but are understudied due to the difficulties in detecting phosphate removal and the lack of good inhibitors. Here the authors develop a light-activated protein phosphatase using photocaged, unnatural amino acids and use it to study ERK nuclear translocation.

## Introduction

Protein phosphorylation is an essential post-translational modification that plays a key role in signal transduction^1^. The transfer of a single phosphate group from ATP to a protein substrate is catalyzed by protein kinases, while the reverse process of removing a phosphate group is catalyzed by protein phosphatases. A typical signaling event results in a cascade of phosphorylations by protein kinases; however, protein phosphatases play an equally important regulatory role in all cell-signaling networks by reversing kinase action through phosphate removal from target proteins^2^. Over the last few decades, several optically controlled protein kinases have been developed and applied in gaining a deeper understanding of the extensive regulatory mechanisms involved in signaling pathways^3–15^; however, optical control of protein phosphatase function has not been achieved^16^. Phosphatases are often difficult to study due to: i) the necessity of detecting a negative signal (removal of a phosphate; which, obviously, needs to be installed first), and ii) the highly conserved, shallow active site, which renders the development of specific small molecule inhibitors nearly impossible^17^.

**Fig. 1:**
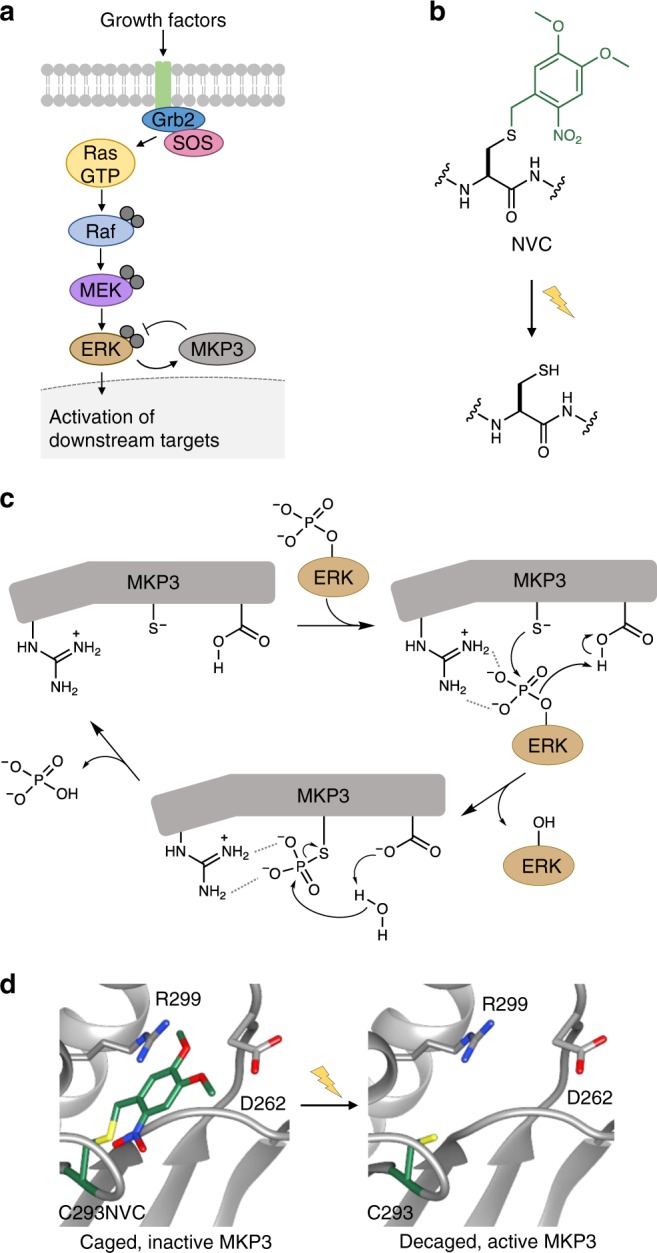
Overview of the MAPK/MKP3 signaling network and application of caged cysteine toward caging the catalytic site of the phosphatase. **a** Schematic of the Ras/Raf/ERK signaling pathway with the dual-specificity phosphatase MKP3 indicated in grey. Upon activation of the pathway by external stimuli, a cascade of phosphorylation events results in dually-phosphorylated ERK, which translocates to the nucleus where it activates additional downstream targets. In the presence of active MKP3, pERK is rapidly dephosphorylated and remains in the cytoplasm. Phosphates are indicated by grey filled circles. **b** Irradiation of NVC removes the nitrobenzyl caging group (green) and restores a native cysteine residue. **c** The mechanism of MKP3-catalyzed ERK dephosphorylation. Dashed lines indicate electrostatic and/or hydrogen-bond interactions. **d** The crystal structure of MKP3 (PDB: 1MKP) is shown with the three critical active site residues labeled in black. On the left, the catalytic cysteine was replaced with NVC, which sterically blocks the active site and prevents catalysis, until exposed to 365 nm light

Mitogen-activated protein kinases (MAPKs) are serine/threonine kinases that convert extracellular stimuli (input) into a range of cellular responses (output). The three conventional MAPK families that have been extensively studied are ERK, JNK, and p38^18^. MAPK signaling is implicated in a number of pathological phenotypes, which generally result from MAPK upregulation or MAPK phosphatase (MKP) down-regulation^19^. MKP3, one of ten MKPs, has high specificity for ERK over JNK or p38^20^. MKP3 is a dual-specificity (it acts on both phosphorylated Thr and Tyr) cytoplasmic phosphatase that recognizes dually-phosphorylated ERK (pERK) and rapidly dephosphorylates it, thus preventing nuclear translocation and activation of downstream targets (Fig. 1a). Aberrant MKP3 levels (both overexpression and down-regulation) have been linked, without a clear mechanistic understanding, to both oncogenic and tumor-suppressive roles in numerous forms of cancer, including pancreatic, lung, colorectal, and thyroid cancer^19,21,22^. Within the same cancer, varying levels of MKP3 expression have been observed, demonstrating the complexity of the role that protein phosphatases play in disease pathology. Thus, an improved understanding of the mechanisms and dynamics of phosphatase-mediated effects on signaling pathways will yield a better understanding of human physiology and disease.

We aim to generate the first light-activated protein phosphatase and select MKP3 as a target due its link to the well-understood substrate pERK. This work lays the foundation to investigate other, less characterized phosphatases through placement under optical control. We envision two distinct approaches for generating a photocaged MKP3: (i) caging of the catalytic cysteine to completely abolish enzymatic activity until photoactivation and (ii) caging of the protein-protein binding interface between MKP3 and ERK2 to abrogate phosphatase-substrate interaction until photoactivation.

## Results

### Optical control of the MKP3 catalytic site

The catalytic domain of MKP3 contains the conserved PTPase sequence HCX_5_R, in which the cysteine acts as a nucleophile in the dephosphorylation reaction of pERK and the arginine interacts with the phosphate group to stabilize the transition state (Fig. 1c). An aspartate facilitates a proton transfer and is essential for efficient catalysis. We hypothesized that replacement of the catalytic cysteine with caged cysteine NVC (Fig. 1b) would mask the nucleophilicity through a thioether bond and block enzymatic activity until UV irradiation removes the caging group (Fig. 1d). Due to the flexibility of the active site pocket, incorporation of NVC is expected to have no effect on protein folding thereby enabling rapid activation of enzymatic function after photolysis.

**Fig. 2:**
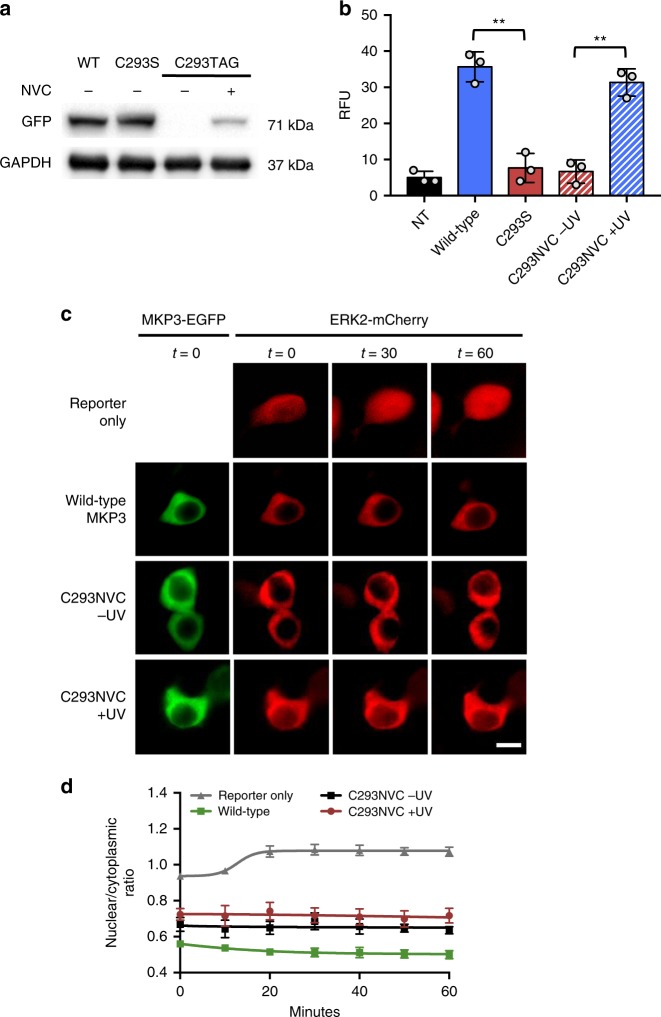
Optical control of the MKP3 catalytic site. **a** Western blot analysis confirms expression of caged MKP3 only in the presence of NVC (1 mM). **b** Biochemical phosphatase assays show complete loss of activity for the caged enzyme, until 365 nm irradiation restores the wild-type enzyme. Error bars denote standard deviation from three biological replicates; **p < 0.01 from unpaired two-tailed Student’s *t*-test. **c** Using an ERK2-mCherry reporter, the activity of wild-type, caged (at the catalytic cysteine), and photoactivated MKP3 was investigated in HEK293T cells. Following pathway stimulation, nuclear exclusion of an ERK2-mCherry reporter is observed for all MKP3 variants. Scale bar 10 μm; also see Supplementary Fig. 6. **d** Quantification of nuclear/cytoplasmic ratios shows successful nuclear translocation for the reporter only control; however, none of the MKP3 variants show any significant change. Error bars represent standard error of the mean from nine individual cells combined from three biological replicates. One-way ANOVA tests at the 60-minute time point results in *p* < 0.0001 for the reporter compared to all MKP3 samples, indicating this a significant difference. Comparing C293NVC –UV to C293NVC + UV results in *p* > 0.1, indicating no significant difference. Data are provided in the Source Data file

We utilized the *E. coli* LeuRS/tRNA pair for genetic encoding of the caged cysteine NVC in human cells and incorporation into MKP3^23^. When transiently expressed with MKP3(C293TAG)-EGFP-HA in HEK293T cells grown in the presence of NVC, full-length MKP3(C293NVC)-EGFP-HA was detected by Western blot (Fig. 2a). In order to validate that the catalytic activity was blocked by the nitroveratryl group, we performed an in vitro biochemical assay. MKP3-EGFP-HA variants were immunoprecipitated from HEK293T cells and phosphatase activity of the immobilized enzymes was measured using the fluorogenic substrate DiFMUP. In the absence of irradiation, the caged sample displayed similar activity to the catalytically dead (C293S) mutant; however, following UV activation, phosphatase enzymatic function was fully restored (Fig. 2b), indicating excellent OFF to ON switching behavior.

To apply our photocaged MKP3 in live cells, we utilized an ERK2-mCherry nuclear translocation reporter (Supplementary Fig. 1a-b)^24^. In a resting cellular state, ERK2 is primarily found in its dephosphorylated form. Upon phosphorylation and activation by upstream kinases, ERK2 translocates to the nucleus. Once in the nucleus, endogenous phosphatases dephosphorylate ERK and the reporter fusion translocates back to the cytoplasm. When HEK293T cells expressed ERK2-mCherry alone, cytoplasmic accumulation of ERK was observed until stimulation with epidermal growth factor (EGF, 100 ng/mL) induced nuclear translocation (Fig. 2c, top row)^24^. Upon co-expression of wild-type MKP3, ERK2 remains in the cytoplasm before and after pathway stimulation with EGF (Fig. 2c, second row)^25,26^. This is a result of the rapid dephosphorylation of pERK2 by MKP3, which prevents any significant nuclear accumulation and thus dampens the EGF signal. Next, we tested whether the active site C293NVC caged MKP3 would have an effect on EGF-induced ERK translocation to the nucleus. When the caged MKP3 and ERK2-mCherry were co-expressed in HEK293T cells, complete cytoplasmic localization was again observed before and after EGF stimulation (Fig. 2c, third row), regardless of UV activation (Fig. 2c, bottom row). The affinities of wild-type and catalytically dead MKP3 for dually-phosphorylated ERK2 are essentially the same (22 and 31 nM, respectively)^27^, thus sequestration of pERK2 by catalytically dead (or catalytically caged) MKP3 anchors pERK2 in the cytoplasm and prevents nuclear translocation regardless of its dephosphorylation activity ( ± UV)^25^. These results indicate that the introduction of a bulky caging group has no effect on the MKP3:ERK2 interaction. Due to the inherent sequestration of ERK2-mCherry by MKP3 variants, we concluded that a different reporter was needed to study the optical triggering of enzymatic dephosphorylation activity in live cells. Additionally, to avoid any potential issues with the imbalance of MEK/ERK signaling components due to the overexpression of ERK2, we sought to utilize a live-cell reporter that monitors endogenous ERK levels^28^. Moreover, these results also indicated that control of the phosphatase:substrate protein-protein interaction may be a second, complementary approach to optically control the cellular function of phosphatases (see below).

**Fig. 3:**
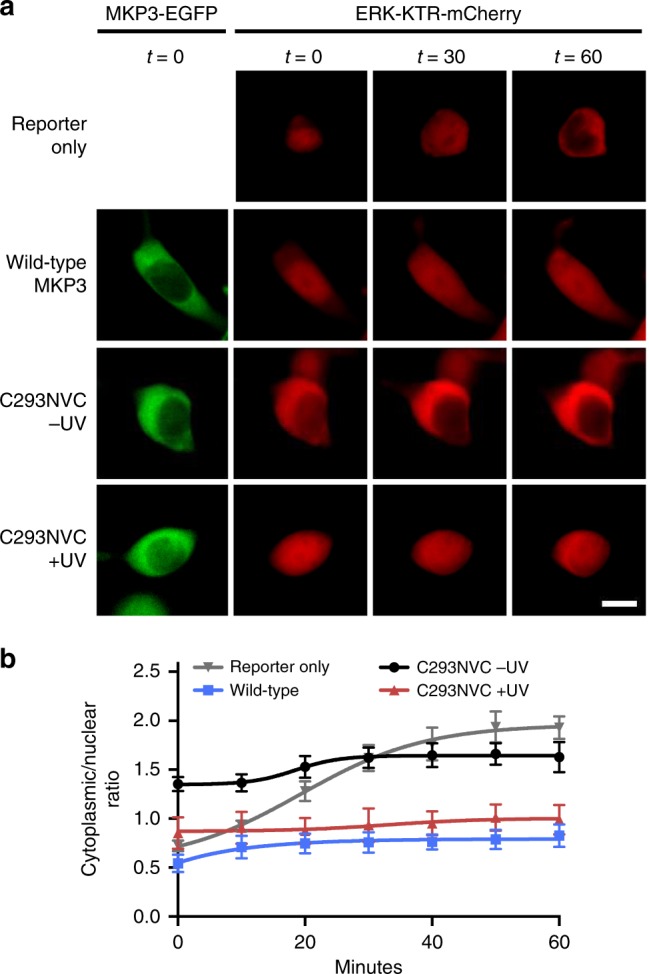
ERK activity in response to optical activation of the MKP3 catalytic site. **a** An ERK-KTR-mCherry reporter was utilized to report on the activity of endogenous ERK2 levels in the presence of wild-type, caged, and photoactivated MKP3 variants in HEK293T cells. Wild-type MKP3 prevents EGF-induced triggering of the reporter, while the caged MKP3 is only active following UV exposure (365 nm, 2 min). Scale bar 10 μm; also see Supplementary Fig. 7. **b** Quantification of the cytoplasmic/nuclear ratio for the MKP3 variants shows successful photoactivation of MKP3. Error bars represent standard error of the mean for nine individual cells combined from three biological replicates. One-way ANOVA tests at the 60-minute time point resulted in *p* < 0.0005 when comparing C293NVC –UV to C293NVC + UV, and reporter only to WT. No significant difference (*p* > 0.1) was observed between WT and C293NVC + UV or reporter only and C293NVC –UV. Data are provided in the Source Data file

Since the binding of MKP3:ERK2 is a dynamic process^2^ and imaging only depicts an average state of ERK2 localization within the cell, we hypothesized that utilization of a reporter that reports on a downstream target of ERK2 (instead of directly detecting ERK2 localization) might enable live-cell monitoring of MKP3 photoactivation. For this, we applied an ERK-KTR-mCherry reporter which utilizes the ERK binding region of Elk-1 fused to a bipartite NLS and NES sequence and is localized in the nucleus in the absence of active pERK, but translocates to the cytoplasm when active pERK is present (see Supplementary Fig. 1c-d for a detailed description)^29^. Importantly, this reporter couples ERK (and pERK) translocation with pERK activity, thus overcoming the inherent limitations of using the direct ERK2-mCherry reporter. HEK293T cells expressing the ERK-KTR-mCherry reporter alone displayed nuclear accumulation prior to EGF addition; however, 60 minutes after pathway stimulation, complete nuclear exclusion occurred (Fig. 3, top row). Upon co-expression of the reporter and wild-type MKP3, we observed maintenance of nuclear localization before and after stimulation (Fig. 3, second row), due to signal dampening through MKP3 dephosphorylation of pERK2. When the active site-caged C293NVC MKP3 was expressed together with the ERK-KTR-mCherry reporter, we observed significant nuclear exclusion following pathway stimulation (Fig. 3, third row). This reflects the dynamic nature of the MKP3:ERK interaction in that pERK is still capable of dissociating from MKP3 and triggering the reporter, while imaging of ERK localization indicates that, on average, the majority of pERK is anchored in the cytoplasm. Upon irradiation of the caged MKP3, nuclear localization of the reporter is maintained, thus demonstrating successful optical control of phosphatase enzymatic function in live cells (Fig. 3, fourth row).

Unlike our results with the ERK2-mCherry reporter, utilization of the ERK-KTR-mCherry reporter allowed for successful demonstration of optical OFF to ON switching of MKP3. While the ERK2-mCherry reporter exclusively measures ERK (or pERK) localization, the ERK-KTR-mCherry construct actually reports on the enzymatic activity of ERK (or pERK). Thus, these two reporters allow for two different functions/outputs to be observed. Additionally, it has been reported that working with physiologically-relevant ERK concentrations is essential for investigating this highly dynamic pathway^28^; therefore, the ERK-KTR reporter presumably reflects biologically relevant findings more accurately. In light of these observations, all remaining experiments were performed with the ERK-KTR-mCherry reporter.

### Optical control of the MKP3 kinase interaction motif

In order to abrogate any potential for substrate sequestration by a caged phosphatase, we next explored the possibility of optically controlling the phosphatase:substrate interface. While optical control of protein active sites through site-specific caging group installation has been used to study a wide range of enzymatic functions (e.g., kinase^3–6^, polymerase^30^, nuclease^31^, recombinase^32^, helicase^33^, and others)^34^, optical control of protein-protein interactions has remained underexplored. In this context of transitioning from optical control of enzymatic function to optical control of protein-protein interactions, caged amino acid mutagenesis represents an advantage over other optogenetic approaches, since the position of the caging group is simply defined by the position of the TAG codon and thus can be readily moved to other sites on the protein, e.g., from the active site to the protein surface.

The kinase interaction motif (KIM) of MKP3 is the region responsible for binding to both ERK and pERK with equal affinity and for conferring a high degree of selectivity for ERK over other MAP kinases. Upon binding of ERK, MKP3 undergoes a conformational change in which a loop in the active site becomes positioned for efficient catalysis; therefore, in the absence of ERK binding, MKP3 catalytic activity is greatly diminished^35^. We hypothesized that caging an essential residue at the MKP3:ERK interface would provide a complementary approach to optically controlling MKP3 function in cells. By blocking the KIM with a light-removable protecting group, both ERK and pERK binding, and thus enzymatic activity and pERK dephosphorylation would be blocked.

**Fig. 4:**
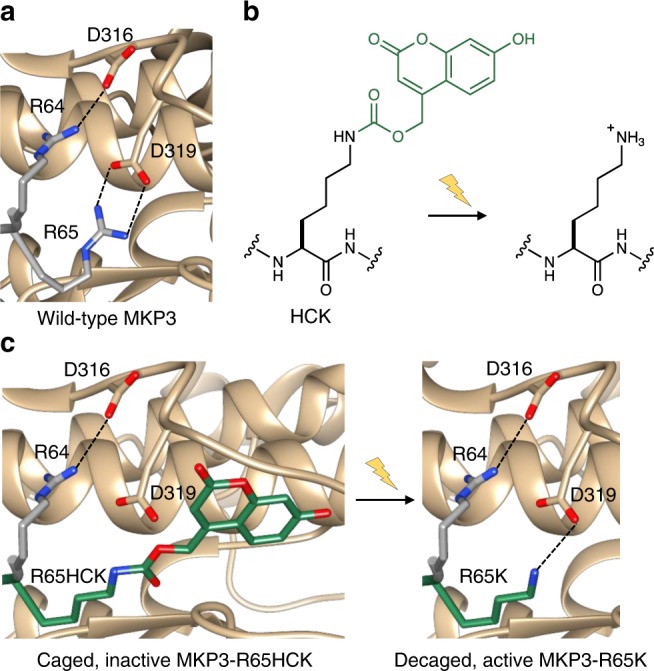
Design strategy for optical control of the phosphatase-kinase interaction motif (KIM). **a** The crystal structure of ERK2 (tan) bound to the KIM peptide of MKP3 (gray) shows essential electrostatic interactions of R64 and R65 with D316 and D319 (PDB: 2FYS). **b** Irradiation of HCK removes the coumarin caging group (green) and restores a native lysine residue. **c** Replacement of R65 with HCK generates steric hindrance and prevents electrostatic interaction until light exposure release K65, which enables the ERK2-MKP3 interaction

For caging the protein-protein interaction, we analyzed the crystal structure of ERK2 bound to the 17 amino acid KIM peptide of MKP3 (residues 60–76)^36^. Two electrostatic interactions between R64 and R65 of MKP3 with D316 and D319, respectively, of ERK2 play a major role in the high affinity binding of these proteins (Fig. 4a). We speculated that optically controlling the electrostatics (positive charge) at either the R64 or R65 site, plus introducing significant steric hindrance, would allow us to optically control the phosphatase:substrate interaction. The photocaged lysine HCK^37^ enables light-induced ammonium ion generation under physiological conditions, since the carbamate greatly reduces the basicity of the ε-amino group, thus providing a potential switch for this protein-protein interaction (Fig. 4b, c).

**Fig. 5:**
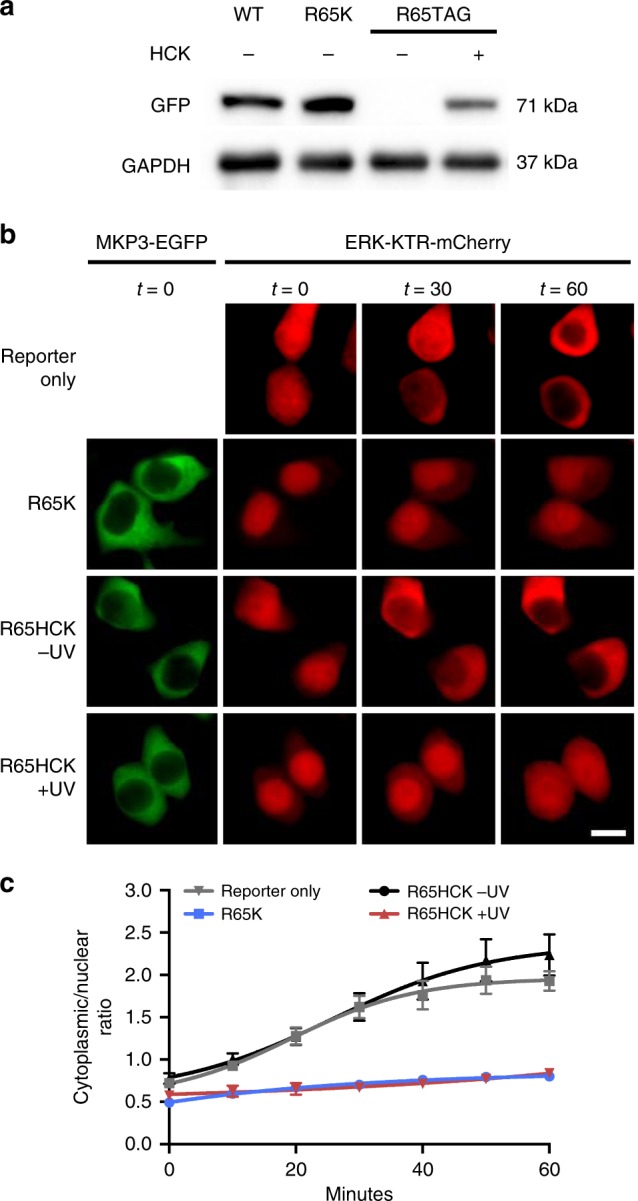
ERK activity in response to optical activation of the MKP3 kinase interaction motif. **a** Western blot of wild-type, R65K, and caged MKP3 shows full-length expression only in the presence of 0.25 mM HCK. **b** KIM-caged MKP3 cannot efficiently bind to and dephosphorylate pERK until UV irradiation (365 nm, 2 min) generates an active enzyme. Scale bar 10 μm; also see Supplementary Fig. 8. **c**) Quantification of cytoplasmic-to-nuclear (C/N) ratios support the micrograph findings. Error bars represent standard error of the mean of nine individual cells from three biological replicates. One-way ANOVA tests at the 60-minute time point resulted in *p* < 0.0001 when comparing R65HCK –UV to R65HCK + UV, and reporter only to R65K. No significant difference (*p* > 0.1) was observed between R65K and R65HCK + UV or reporter only and R65HCK –UV. Data are provided in the Source Data file

It has been reported that replacement of R64 with lysine had no effect on ERK2 binding affinity; however, an R65K mutation resulted in a 50-fold lower affinity for ERK2 compared to wild-type MKP3^38^. Interestingly, almost identical MKP3 phosphatase activity was reported for both mutants. Based on these results, we tested both the R64K and R65K mutants using the ERK-KTR-mCherry reporter in HEK293T and compared their activity to wild-type MKP3. Not surprisingly, both lysine mutants showed similar phosphatase activity (Supplementary Fig. 2) and were only slightly less active than wild-type in the KTR reporter assay. Since R65 is reported to be the more critical residue for ERK2 binding, we incorporated HCK at that position (Fig. 5a). We again utilized the ERK-KTR-mCherry reporter for exploring photoactivation of the caged KIM. HEK293T cells expressing only the reporter showed complete nuclear exclusion after EGF stimulation (Fig. 5b, first row), while cells co-expressing the R65K mutant led to mostly nuclear reporter accumulation, as expected (Fig. 5b, second row). When the reporter and KIM-caged R65HCK MKP3 were co-expressed, we observed complete nuclear exclusion following EGF stimulation, indicating inactivity of the phosphatase (Fig. 5b, third row). Upon irradiation and activation of the caged MKP3, nuclear localization of the reporter is maintained, thus demonstrating that the cellular function of the phosphatase, specifically its ability to engage its substrate, was optically triggered (Fig. 5b, fourth row). Quantification of the micrograph fluorescence clearly displays the ability to optically control the ERK2-MKP3 interaction via caging of the phosphatase KIM, thereby enabling light-triggered substrate binding and dephosphorylation (Fig. 5c). As in the case of active site cysteine caging, introduction of a caging group into the phosphatase:substrate interface also provides excellent optical OFF to ON switching behavior.

### Tunability of optically controlled MKP3

**Fig. 6:**
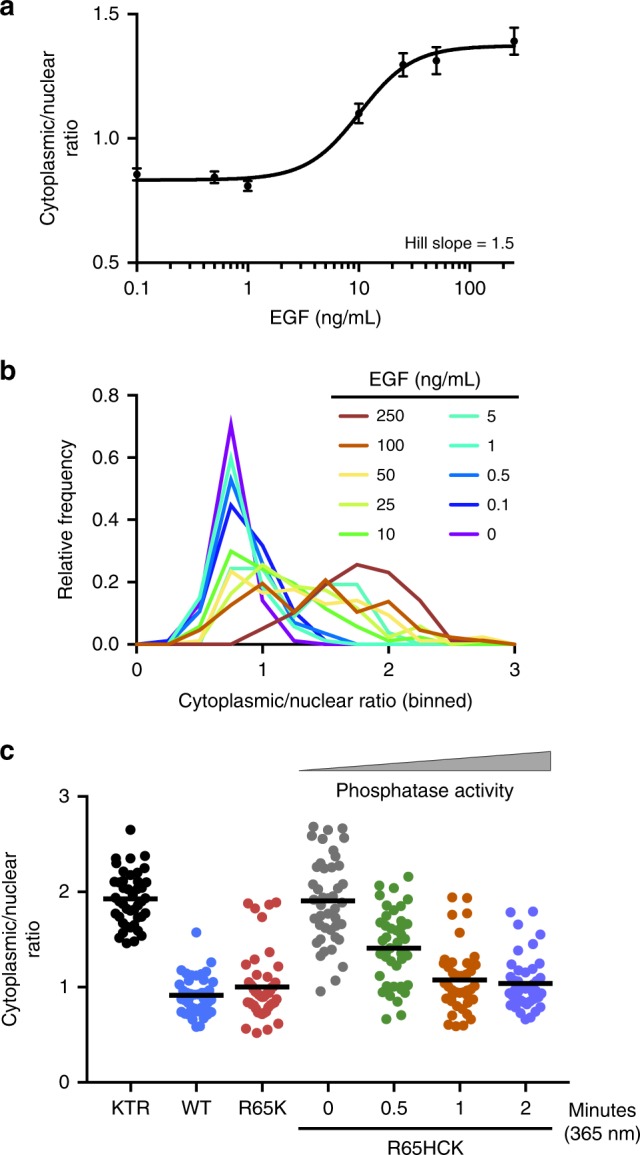
Analysis of ERK activity in response to different doses of growth factor stimulation and optically tuned phosphatase function. **a** The cytoplasmic/nuclear ratios for the ERK-KTR-mCherry reporter in HEK293T cells were plotted for a range of EGF concentrations and fitted with a Hill function. Based on the calculated Hill slope, a graded response is observed. Error bars represent the standard error of the mean of 85–90 cells per condition pooled from three biological replicates. **b** Histogram representation of the obtained data more clearly depicts the graded response as seen by a unimodal distribution. **c** Optical activation of KIM-caged MKP3 shows a graded response for the regulation of pERK translocation upon increased UV irradiation and cell surface stimulation with EGF (100 ng/mL), and thus increased levels of active MKP3. Each dot represents a single cell, black lines represent the mean, *n* = 40–45. Quantified cells were pooled from three biological replicates. One-way ANOVA tests showed *p* < 0.0001 comparing 0 min to 0.5 min irradiation, *p* = 0.0002 for 0.5–1 min irradiation, and *p* > 0.1 for 1–2 min irradiation, indicating a significant difference for the first two comparisons and no significant difference for the third. Data are provided in the Source Data file

After demonstrating that we could successfully photo-activate the MKP3:ERK interaction and subsequently control phosphatase activity, we sought to explore the effect of titrating the level of active versus inactive phosphatase in order to further characterize the role of MKP3 in downregulating the ERK pathway. It is well established that ERK phosphorylation occurs in a graded manner, meaning pERK levels increase almost proportionally to an increase in external stimulus^39,40^. However, downstream targets of the ERK pathway often act in a switch-like manner, meaning there is an all-or-nothing response. This has been reported for the nuclear translocation of ERK2 monitored as an ERK2-EGFP fusion in both HeLa and PC12 cells^41^. We utilized the ERK-KTR-mCherry reporter to characterize ERK nuclear translocation behavior in HEK293T cells and observed a graded response upon varied EGF stimulation (Fig. 6a, b). For classification as a graded or switch-like response, the cytoplasmic/nuclear ratio for increasing EGF concentrations was fitted with a Hill function to calculate the Hill slope. A slope of approximately 1 indicates a graded response, while a slope of »1 is representative of switch-like behavior^41^. A slope of 1.5 was calculated, thus indicating a graded response. Unlike the monitoring with an ERK2-GFP fusion which only reports protein localization, this reporter couples nuclear translocation with reporting of nuclear activity of pERK. Our results showcase the complexity and variability of the ERK signaling pathway within different cell types. We were curious how titrating MKP3 phosphatase levels would affect the ERK activity response in the KTR reporter. We observed a graded reporter response to increasing MKP3 activity upon increasing UV exposure (Fig. 6c). Almost complete activation is achieved with a two-minute irradiation, thus irradiation for shorter times is expected to allow for titration of increasing levels of active versus inactive phosphatase. With shorter irradiation times, a partial response was observed where only a small amount of pERK is dephosphorylated and results in incomplete exclusion of the reporter. This graded response indicates that MKP3 functions to dampen the response to growth factor stimulation like a rheostat rather than completely blocking signal propagation above a certain threshold like a switch. The MKP family has been considered more of a modulator of MAPK signal intensity and duration, rather than an ‘off switch’^2^, thus the graded response observed by us supports those previous hypotheses.

These results represent the first example of photoactivation of a protein phosphatase in live cells. Unlike traditional methods employed for studying phosphatases (e.g., gene knockdown), the optogenetic strategies developed here allow for acute triggering of enzyme activity, generating tunable levels of activity depending on the length of light exposure, and temporal control of activation. Two complementary approaches were developed in order to render the dual-specific phosphatase MKP3 light-activatable, thereby enabling independent optical control of catalytic function and of phosphatase-substrate interaction. In the first approach, incorporation of caged cysteine into the active site enabled direct control of catalytic activity by blocking an essential catalytic residue. Based on the extensive active site conservation of dual-specificity phosphatases, this approach is generalizable and can be used to optically control other members of the DUSP family. In addition to DUSPs, most tyrosine phosphatases utilize a catalytic cysteine, enabling expansion of our strategy to many of the 200 protein phosphatases. In addition, we applied a second approach by strategic caging of a lysine-mediated electrostatic interaction in the MKP3:ERK protein-protein interface. Optical control of the kinase interaction motif will be useful for placing other phosphatase-MAPK interactions under optical control since electrostatic interactions are most often the main determinants of these high affinity interactions. More broadly, light-regulation of catalytic sites through introduction of caged amino acids has been demonstrated numerous times; however, the use of caged amino acids to control protein-protein interactions remains underdeveloped^6,42–44^ and extension of this approach to other scaffolding proteins will further facilitate the optical dissection of cell-signaling networks.

## Methods

### Reagents

The photocaged amino acids NVC and HCK were synthesized as previously reported^23,37^. Reagents used for synthesis were obtained from commercial vendors and used without additional purification. Recombinant animal-free human epidermal growth factor (EGF) was purchased from PeproTech (AF-100-15) and reconstituted per the manufacturer’s recommendation. Live Cell Imaging Solution (LCIS) was purchased from Molecular Probes/Invitrogen (A14291DJ). All antibodies used in Western blots and immunoprecipitations were purchased from ProteinTech: GFP (50430-2-AP), GAPDH (10494-1-AP), and goat anti-rabbit HRP (SA00001-2).

### DNA constructs

All cloning was performed using chemically-competent Top10 cells (Invitrogen).

The synthetase plasmid (pMAH2-CageCys) used for caged cysteine (NVC) incorporation was a gift from Hui-wang Ai (UVA)^23^. The MKP3 gene was PCR-amplified using primers P1 and P2 from Addgene plasmid #27975 and ligated into EGFP-N1 (Clontech 6085-1) using restriction sites HindIII and BamHI to generate pMKP3-EGFP. Plasmid sequence was confirmed by Genewiz Sanger sequencing with their CMV-forward primer. An HA epitope tag was added to the C-terminus of the MKP3-EGFP fusion using primers P3 and P4 following a site-directed mutagenesis method^45^ with pMKP3-EGFP as the template to yield pMKP3-EGFP-HA which was confirmed by sequencing at Genewiz with their SV40 polyA reverse primer.

To improve incorporation levels for caged cysteine, the MKP3-EGFP-HA coding sequence was PCR amplified with primers P5 and P6 and ligated into the pMAH vector following digestion with SacI and PmeI to remove the synthetase (doubling the number of tRNAs genes in a given experiment), thus generating pMAH-MKP3-EGFP-HA-WT. Site-directed mutagenesis^45^ was performed to generate pMAH-MKP3-EGFP-HA-C293TAG (primers P7 and P8) and pMAH-MKP3-EGFP-HA-C293S (primers P9 and P10) mutations using the pMAH-MKP3-EGFP-HA-WT construct. All pMAH constructs were confirmed by Sanger sequencing (Genewiz) using their CMV-forward primer and BGH polyA reverse primer.

The pMKP3-EGFP-HA construct was used for generating pMKP3-EGFP-HA-R65K (primers P11 and P12), pMKP3-EGFP-HA-R65TAG (primers P13 and P14), and pMKP3-EGFP-HA-R64K (primers P15 and P16) mutants via site-directed mutagenesis^45^. Sanger sequencing (Genewiz) was performed to confirm successful mutagenesis with their CMV-forward primer. Two PylRS HCKRS/tRNA vectors were utilized: pU6-PylT-H1-PylT-HCKRS and p(U6-PylT)_x4_-HCKRS^46^. Due to the significant difference in size of these two plasmids, the smaller plasmid, pU6-PylT-H1-PylT-HCKRS, was used in live-cell imaging experiments as triple transfections were required, while the larger plasmid, p(U6-PylT)_x4_-HCKRS, was used for western blot analysis after double transfections.

The pERK2-mCherry reporter was a gift from Jason Haugh (NCSU)^47^. The pERK-KTR-mCherry reporter was generated by PCR amplifying the ERK-KTR fragment from Addgene plasmid #59150 with primers P17 and P18 and the backbone fragment was generated by PCR amplifying from an mCherry-N1 construct (gift from Gerry Hammond, University of Pittsburgh) with primers P19 and P20. The two fragments (ERK-KTR and mCherry) underwent Gibson assembly^48^ to generate pERK-KTR-mCherry. Plasmid sequence was confirmed by Genewiz using their CMV-forward primer.

For a list of all primer sequences, see Supplementary Table 1. For maps of all plasmids, see Supplementary Fig. 3.

### General cell culture and transfection

HEK293T cells (ATCC, CRL-11268) were maintained at 37 °C in 5% CO_2_ atmosphere in DMEM High Glucose with 4.5 mM L-glutamine (GE Life Sciences, SH30003.03) supplemented with 1 mM sodium pyruvate (Alfa, A11148), 10% fetal bovine serum (Sigma, F0926), and 1% penicillin-streptomycin (Corning, 30-002-CI). For a 96-well format used in all imaging-based experiments, 100 µL of media was used for culturing, whereas for a 12-well plate used for western blot or biochemical assays, 1 mL of media was used. Cells were monitored every 3 months to confirm the absence of mycoplasma contamination (Genlantis, MY01100). Cells were transiently transfected with LPEI (Polysciences, 23966) using a 0.5 mg/mL solution at a 5:1 LPEI:DNA (w/w) ratio in antibiotic-free DMEM. Following overnight transfection, the media was removed and the cells were gently washed once with regular DMEM to remove any excess unnatural amino acid. Cells were serum-starved (DMEM with 0.1% FBS, without antibiotics) for 4 h. The media was replaced with 90 µL of LCIS immediately prior to irradiation. Irradiation was performed on a UV transilluminator (VWR Dual Transilluminator at 365 nm) for two minutes (unless specified otherwise).

### General live-cell imaging

HEK293T cells were imaged at ambient temperature with an Axio Observer Z1 microscope using a 20x Plan Apochromat objective equipped with an AxioCam MRm camera using Zen 2 Blue Edition software. Growth factor stimulation was performed by diluting a 10 × (1 µg/mL) stock directly into media containing cells on the microscope stage to a final concentration of 100 ng/mL. Images were acquired using EGFP and mCherry filter sets (Zeiss 38HE and 43HE, respectively). All image analysis and quantification was performed using FIJI and specific details are provided in the corresponding sections.

### Expression of MKP3 with a caged catalytic site

To validate the expression of wild-type, catalytically dead, and caged MKP3 via western blot, HEK293T cells were plated at ~ 200,000 cells per well in a 12-well clear bottom plate (Greiner). At ~ 80% confluence, cells were transfected as follows: (1) 1000 ng of pMAH-MKP3-EGFP-HA-WT and 1000 ng of pMAH2-CageCys, (2) 1000 ng of pMAH-MKP3-EGFP-HA-C293S and 1000 ng of pMAH2-CageCys, or (3) 1000 ng of pMAH-MKP3-EGFP-HA-C293TAG and 1000 ng of pMAH2-CageCys with 20 µL of LPEI using 100 µL of OptiMEM. The transfection mix (OptiMEM, DNA, and LPEI) was incubated at room temperature for 20 min, then the entire volume was added to wells containing 1 mL of DMEM (antibiotic-free). For NVC-containing wells, 10 µL of NVC stock solution (100 mM in DMSO) were diluted into 990 µL of DMEM for a final 1 mM NVC concentration. Cells were incubated for ~ 24 h prior to harvesting for western blot analysis (see next section).

### Western blot analysis

Proteins were expressed in HEK293T cells as described (see Expression of MKP3 with a caged catalytic site or Expression of MKP3 with a caged kinase interaction motif) and the 12-well plate was placed on ice, the media was removed, and the cells were washed with 200 µL of ice-cold phosphate buffer saline (PBS), then lysed with 100 µL of ice-cold RIPA lysis buffer (150 mM NaCl, 1.0% NP-40, 0.5% sodium deoxycholate, 0.1% SDS, 50 mM Tris pH 8.0) supplemented with 1 × protease inhibitor cocktail (Thermo Scientific, 78429). Cellular debris was pelleted at 21,000 g for 15 min at 4 °C and the supernatant was collected and mixed with 4 × Laemmli sample buffer (12 µL lysate + 4 µL 4 × buffer). Samples were heated at 95 °C for 10 min, then frozen at –80 °C. The samples (all 16 µL per condition) were resolved on a 10% SDS-PAGE (60 V for 20 min, 150 V for 1.25 h), then transferred to a 0.45 µm PVDF membrane (Millipore, IPVH00010) at 80 V for 1.5 h using Towbin buffer (25 mM Tris-HCl, 192 mM glycine, pH 8.3, 20% methanol [v/v]). The membrane was blocked for 2 h with 5% milk in TBST (0.1% Tween-20 in 1 × TBS) at room temperature with rocking. After blocking, the membrane was cut in half horizontally. The top half was probed with anti-GFP, while the bottom half was probed with anti-GAPDH. Anti-GFP was diluted 1:5000 and anti-GAPDH was diluted 1:5000 in 5% milk in 6 mL of TBST and the membranes were incubated with primary antibodies overnight with rocking at 4 °C. The following day, membranes were washed thrice with TBST (~ 10 mL), then probed with goat anti-rabbit HRP using 1:10000 dilution in 6 mL of TBST for 2 h. The membranes were again washed thrice with TBST (~ 10 mL). Membranes were developed with SuperSignal West Pico PLUS Chemiluminescent Substrate (Thermo Scientific, 34580) and imaged on a BioRad ChemiDoc using the Chemi setting. Images were exported using the “auto scale” feature in the BioRad Image Lab software. Uncropped/unprocessed western blot images are included in the Source Data file.

### Biochemical phosphatase assays

MKP3 variants were expressed in a 12-well format identical to the method used above for western blot analysis. The plate was chilled on ice, the media was removed, and cells were gently washed with 1 mL of ice-cold PBS. Cells were gently lysed with 100 µL of GE Mammalian Protein Extraction Buffer (28941279) on ice. The plate (still on ice) was placed on a rotary shaker and shaken at 250 rpm for 15 min. The lysate was collected, and cellular debris was pelleted at 21,000 g for 10 min at 4 °C. The supernatant (~90 µL to avoid disturbing the pellet) was collected into a fresh tube. Anti-GFP antibody was added to each sample (0.1 µg) and incubated for 1 h at 4 °C. Protein A resin (SCBT, sc-2001) was added to each sample (10 µL) and rocked for 2 h in a cold room. The resin was gently pelleted at 100 g for 5 min at 4 °C. The supernatant was removed (and discarded) and the resin was washed twice (500 µL each time) with chilled phosphatase assay buffer (25 mM HEPES, 50 mM NaCl, 2.5 mM EDTA, 10 mM DTT, pH 7.2). To activate MKP3 for in vitro assays, 5 µg of purified GST-ERK2 was added to each sample (see below for its expression in *E. coli*). DiFMUP (Invitrogen, D6567) was added to a final concentration of 50 µM (from a 10 mM stock prepared in DMSO) and reactions were incubated at 37 °C for 30 min. Fluorescence was measured using a Tecan M1000 pro with settings of 358/5 nm for excitation and 450/5 nm for emission. Background hydrolysis was measured in buffer plus DiFMUP and was subtracted from all data points. Expression and immunoprecipitation was performed in biological triplicates and fluorescence readings were averaged with error bars representing standard deviations.

To generate GST-ERK2 protein, Addgene plasmid pGEX-4T-1 3xFlag-ERK2 (#47573) was transformed into BL21(DE3) using LB broth supplemented with ampicillin (100 µg/mL) and protein expression was performed following a literature procedure at a 100 mL scale^49^. In short, a 100 mL LB culture (plus ampicillin) was prepared by inoculating with 1 mL of saturated overnight culture, then grown to OD_600_ of 0.6 at 37 °C with shaking. At this point, expression was induced by the addition of IPTG (using a 1 M stock solution prepared in water) to a final 2 mM concentration, and the culture was shaken for 5 h at 37 °C. Cells were harvested by centrifugation at 4200 *g* at 4 °C for 10 min, and purification commenced by resuspending the cell pellet in 20 mL of ice-cold PBS containing 1 × protease inhibitor (Sigma, P8849). Cell lysis was performed using an EmulsiFlex C3 Homogenizer (Avestin). Cells were passed through without pressure for 5 min, then cycled through with pressure (20,000 psi) for 15 min. Cellular debris was pelleted at 21,000 *g* for 15 min at 4 °C, and the soluble protein fraction was transferred to a fresh tube. GST-ERK2 was purified using Pierce Glutathione Agarose (PI16100) following the manufacturer’s protocol. Purified protein was buffer exchanged thrice using centrifuge filters (Amicon Ultra 10 kDa, 0.5 mL) into phosphatase assay buffer prior to use in the in vitro assays and diluted to a final concentration of 1 µg/µL. Protein concentration was determined using a Coomassie-stained SDS-PAGE gel with a BSA standard curve.

### Catalytic site-caged MKP3 and ERK2-mCherry reporter

HEK293T cells were plated at ~50,000 cells per well in a black, poly-D-lysine coated 96-well clear bottom plate (Greiner). At ~ 80% confluence, cells were transfected as follows: (1) 75 ng of pERK2-mCherry and 300 ng of pMAH2-CageCys, (2) 75 ng of pERK2-mCherry, 150 ng of pMAH-MKP3-EGFP-HA-WT, and 150 ng of pMAH2-CageCys, or (3) 75 ng of pERK2-mCherry, 150 ng of pMAH-EGFP-HA-C293TAG, and 150 ng of pMAH2-CageCys using 3.75 µL of LPEI (0.5 mg/mL) in 20 µL of OptiMEM. Transfection mix was added to wells containing 100 µL of DMEM. For NVC-containing wells, DMEM was prepared as above to yield 1 mM NVC. Cells were incubated with the transfection mix for ~24 h prior to serum starving. Cells were serum-starved (DMEM with 0.1% FBS, without antibiotics) for 4 h. The media was replaced with 90 µL of LCIS and the cells were irradiated for 2 min with a UV transilluminator (from the bottom of the plate), then immediately placed on the microscope stage for fluorescence imaging. EGF activation was performed as described previously (see General live-cell imaging) using a final concentration of 100 ng/mL. Images were acquired every 10 min for 60 min using GFP and mCherry filters.

Circular ROIs were selected using the EGFP channel in order to discriminate between nuclear and cytoplasmic signal. Mean fluorescence was used for determining nuclear/cytoplasmic (N/C) ratios. For comparison of the MKP3 mutants over time, nine cells were analyzed at each time point from 0 to 60 from the three biological replicates and the average values +/– standard error of the mean were plotted against time. One-way ANOVA tests were performed to evaluate the statistical significance of the data.

### Catalytic site-caged MKP3 and ERK-KTR-mCherry reporter

HEK293T cells were plated at ~ 50,000 cells per well in a black, poly-D-lysine coated 96-well clear bottom plate. At ~80% confluence, cells were transfected as follows: (1) 150 ng of pERK-KTR-mCherry and 300 ng of pMAH2-CageCys, (2) 150 ng of pERK-KTR-mCherry, 150 ng of pMAH-MKP3-EGFP-HA-WT, and 150 ng of pMAH2-CageCys, or (3) 150 ng of pERK-KTR-mCherry, 150 ng of pMAH-MKP3-EGFP-HA, and 150 ng of pMAH2-CageCys using 4.5 µL of LPEI in 20 µL of OptiMEM. Transfection mix was added to wells containing 100 µL of DMEM. For NVC-containing wells, DMEM was prepared as above for 1 mM final concentration. Cells were incubated with the transfection mix for ~ 24 h prior to serum starving. Serum starvation, light activation, EGF stimulation, and image acquisition were performed identical to section Catalytic site-caged MKP3 and ERK2-mCherry reporter.

Circular ROIs were selected using the EGFP channel in order to discriminate between nuclear and cytoplasmic signal. Mean fluorescence was used for determining cytoplasmic/nuclear (C/N) ratios. For comparison of the MKP3 mutants over time, nine cells were analyzed at each time point from 0 to 60 from the three biological replicates and the average values +/– standard error of the mean were plotted against time. One-way ANOVA tests were performed to evaluate the statistical significance of the data.

### Expression of MKP3 with a caged kinase interaction motif

To validate the expression of wild-type, R65K, and caged MKP3 via western blot, HEK293T cells were plated at ~200,000 cells per well in a 12-well clear bottom plate. At ~80% confluence, cells were transfected as follows: (1) 1000 ng of pMAH-MKP3-EGFP-HA-WT and 1000 ng of p(U6-PylT)_x4_-HCKRS, (2) 1000 ng of pMKP3-EGFP-HA-R65K and 1000 ng p(U6-PylT)_x4_-HCKRS, or (3) 1000 ng of pMKP3-EGFP-HA-R65TAG and 1000 ng of p(U6-PylT)_x4_-HCKRS with 20 µL of LPEI using 100 µL of OptiMEM. The transfection mix (OptiMEM, DNA, and LPEI) was incubated at room temperature for 20 min, then the entire volume was added to wells containing 1 mL of DMEM (antibiotic-free). For HCK-containing wells, 2.5 µL of HCK (100 mM stock in DMSO) was diluted in 998 µL of DMEM for a final 0.25 mM HCK concentration. Cells were incubated for ~24 h prior to harvesting for western blot analysis (see section above).

### KIM-caged MKP3 and ERK-KTR-mCherry reporter

HEK293T cells were plated at ~50,000 cells per well in a black, poly-D-lysine coated 96-well clear bottom plate. At ~80% confluence, cells were transfected as follows: (1) 150 ng of pERK-KTR-mCherry and 300 ng of pU6-PylT-H1-PylT-HCKRS, (2) 150 ng of pERK-KTR-mCherry, 150 ng of pMKP3-EGFP-HA-R65K, and 150 ng of pU6-PylT-H1-PylT-HCKRS, (3) 150 ng of pERK-KTR-mCherry, 150 ng of pMKP3-EGFP-HA-R65TAG, and 150 ng of pU6-PylT-H1-PylT-HCKRS using 4.5 µL of LPEI in 20 µL of OptiMEM. Transfection mix was applied to wells containing 100 µL of DMEM. HCK in DMEM was prepared as above and 100 µL was added per well. Serum starvation, light activation, EGF stimulation, and image acquisition were performed identical to section Catalytic site-caged MKP3 and ERK2-mCherry reporter.

Circular ROIs were selected using the EGFP channel in order to discriminate between nuclear and cytoplasmic signal. Mean fluorescence was used for determining cytoplasmic/nuclear (C/N) ratios. For comparison of C/N ratios over time for the MKP3 mutants, nine cells were analyzed at each time point from 0 to 60 from the three biological replicates and the average values ± standard error of the mean were plotted against time. One-way ANOVA tests were performed to evaluate the statistical significance of the data.

### Tunability of ERK activity

HEK293T cells were plated at ~50,000 cells per well in a black, poly-D-lysine coated 96-well plate. At ~80% confluence, cells were transfected with 150 ng of pERK-KTR-mCherry using 1.5 µL of LPEI (0.5 mg/mL) in 20 µL of OptiMEM. Transfection mix (OptiMEM, DNA, and LPEI) was incubated at room temperature for 20 min, then the entire volume was added to wells containing 100 µL of DMEM (antibiotic-free). Cells were incubated with the transfection mix for ~24 h prior to serum starvation. Cells were starved for 4 h in DMEM with 0.1% FBS (without antibiotics), then the media was replaced with 90 µL of LCIS. A range of EGF concentrations (from 2500 ng/mL to 1 ng/mL, for final range of 250 ng/mL to 0.1 ng/mL after 1:10 dilution upon addition to cells) was generated starting with a 10 µg/mL stock and diluting into LCIS. Following EGF addition, a 60-minute time point image was acquired for all ten EGF concentrations.

Circular ROIs were used to measure mean fluorescence in the nucleus and cytoplasm. C/N ratios were calculated for 85–90 cells per condition (combined from three biological replicates) and the averages ± standard error of the mean (s.e.m.) were plotted. A curve was fitted using the settings “[agonist] versus response – variable slope (four parameters)” in GraphPad Prism 7. The Hill slope was computed to be 1.534.

### Tunability of KIM-caged MKP3 activity

For exploring the tunability of photoactivation, irradiation times of 30, 60, and 120 s were utilized on cells transfected as described above (section KIM-caged MKP3 and ERK-KTR-mCherry reporter). Irradiation was performed as previously described (see General cell culture and transfection) and cells were immediately placed on the microscope stage for imaging. EGF activation was performed as described previously using 100 ng/mL final concentration. A 60-minute time point image was acquired.

Circular ROIs were selected using the EGFP channel in order to discriminate between nuclear and cytoplasmic signal. Mean fluorescence was used for determining cytoplasmic/nuclear (C/N) ratios. C/N ratios were determined for 40–45 cells per condition from a combined three biological replicates. A dot plot representation shows the C/N for each of the analyzed cells and a horizontal bar indicates the average. Histogram representation used a bin range of 0 to 3 with a bin size of 0.25. One-way ANOVA tests were performed to evaluate the statistical significance of the data.

### Effect of UV irradiation on cell-signaling reporters

HEK293T cells were plated at ~50,000 cells per well in a black, poly-D-lysine coated 96-well clear bottom plate. At ~80% confluence, cells were transfected with either: (1) 150 ng of pERK-KTR-mCherry or (2) 75 ng of pERK2-mCherry using 1.5 µL or 0.75 µL of LPEI, respectively, in 20 µL of OptiMEM (note: a working solution of transfection mix was prepared for four wells in a single tube). Transfection mix was applied to wells containing 100 µL of DMEM. Cells were incubated with the transfection mix for ~24 h prior to serum starving. Cells were starved for 4 h in DMEM with 0.1% FBS (without antibiotics), then the media was replaced with 90 µL of LCIS. Following starvation and media change, a subset of wells was irradiated for 2 min with a UV transilluminator, then immediately placed on the microscope stage for fluorescence imaging. EGF activation was performed as described previously using 100 ng/mL final concentration (a subset of wells was left unstimulated). Images were acquired every 10 minutes for 60 minutes. Mean fluorescence from circular ROIs was used for determining cytoplasmic/nuclear (C/N) ratios for the ERK-KTR reporter or nuclear/cytoplasmic (N/C) ratios for the ERK2-mCherry reporter. Nine cells were analyzed at each time point from 0 to 60 minutes combined from three biological replicates and the average values +/– standard error of the mean were plotted against time. One-way ANOVA tests were performed to evaluate the statistical significance of the data. Data are presented in Supplementary Fig. 4a–b.

### Effect of UV irradiation on cell viability

HEK293T cells were plated at ~25,000 cells per well in a white, poly-D-lysine coated 96-well clear bottom plate. The next day, four different treatments were performed in triplicate: (1) non-treated, (2) 100 µM doxorubicin, (3) UV irradiation for 2 min, or (4) 0.1% DMSO, all in regular DMEM. Cells were maintained at 37 °C and 5% CO_2_ atmosphere for 72 h. An XTT cell viability assay^50^ was performed by adding 40 µL of the activated XTT reagent (8 µL of 1.7 mg/mL menadione diluted into 1 mL of 1 mg/mL XTT reagent solution) to each well. Absorbance was measured at 450 nm and 630 nm (background) on a Tecan M1000 pro plate reader immediately following reagent addition. Cells were placed back in the incubator for four hours, then final absorbance measurements were taken. The background absorbance was subtracted from each well, then absorbance was normalized such that the non-treated sample equaled 100% cell viability. Data are presented in Supplementary Fig. 5.

### Reporting summary

Further information on research design is available in the Nature Research Reporting Summary linked to this article.

## Supplementary information

Supplementary Information

Reporting Summary

Source Data

## Data Availability

The source data that support the findings of Figs. 2a, 2b, 2d, 3b, 5a, 5c, 6a-c, and the Supplementary Figs. 2, 4a-b, and 5 are included in the Source Data file. Any additional data are available from the corresponding author upon reasonable request.
