# Supplementary material for: Optical control of protein phosphatase function
Source: Nat Commun. 2019 Sep 26;10:4384. doi: 10.1038/s41467-019-12260-z (PMC6763421; doi:10.1038/s41467-019-12260-z)
Supplement: Supplementary file 1 — Supplementary Information [file 41467_2019_12260_MOESM1_ESM.pdf]

SUPPLEMENTARY INFORMATION

Optical Control of Protein Phosphatase Function

Taylor M. Courtney and Alexander Deiters*

Department of Chemistry, University of Pittsburgh, Pittsburgh, PA 15260, United States

deiters@pitt.edu

Supplementary Figures:

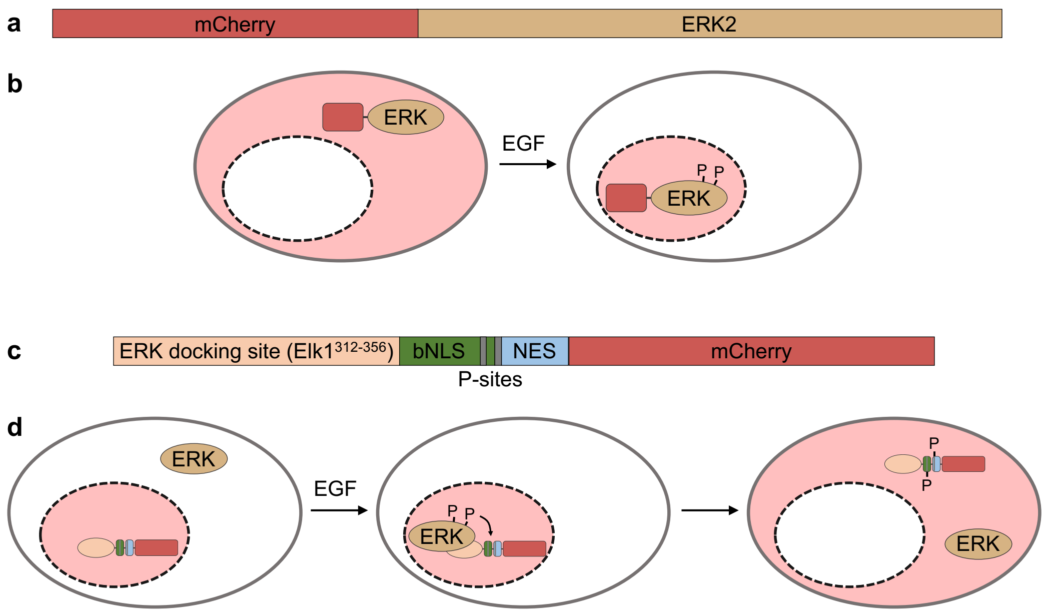

Supplementary Figure 1. Schematic depictions of the two live cell reporters used for imaging experiments. a) Gene sequence of the ERK-mCherry reporter. b) When expressed in cells, ERK-mCherry resides primarily in the cytoplasm in the absence of growth factor stimulation (inactive MAPK signaling); however, following activation with EGF, ERK is phosphorylated and translocates to the nucleus. c) Gene sequence of the ERK-KTR-mCherry reporter.[1] The ERK docking site of the transcription factor Elk1 (residues 312 – 356) is fused to a bipartite nuclear localization sequence (bNLS) and a nuclear export sequence (NES), followed by mCherry. The two “P-sites” indicate residues in the NLS and NES which are phosphorylated by pERK. d) Prior to stimulation of the MAPK/ERK pathway with external growth factors, the reporter resides in the nucleus. Upon ERK phosphorylation, pERK translocates to the nucleus where it binds the docking domain and phosphorylates both P-sites. Phosphorylation of the reporter represses the nuclear localization signal and activates the nuclear export sequence, resulting in transport of mCherry to the cytoplasm. Unlike other FRET-based kinase reporters (e.g., EKARs), the KTR technology requires only a single fluorescent protein, has excellent dynamic range, and can be activated/deactivated faster.

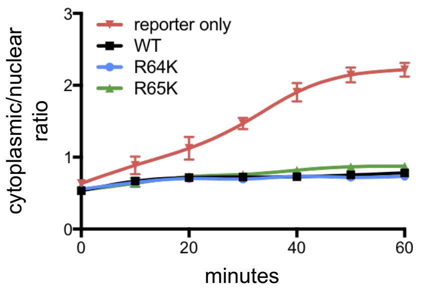

Supplementary Figure 2. Co-expression of wild-type MKP3, R64K MKP3, or R65K MKP3 with the ERK-KTR-mCherry reporter demonstrates that both R64K and R65K closely mimic the activity of the wild-type phosphatase. The cytoplasmic/nuclear ratio over time for the four conditions shows significant nuclear exclusion in the case of the reporter alone; however, upon co-expression of wild-type (WT), R65K, or R64K MKP3, minimal change in reporter localization is observed due to similar levels of phosphatase activity under all three conditions. Error bars indicate standard deviation of five individual cells per condition from two biological replicates. Data are provided in the Source Data file.

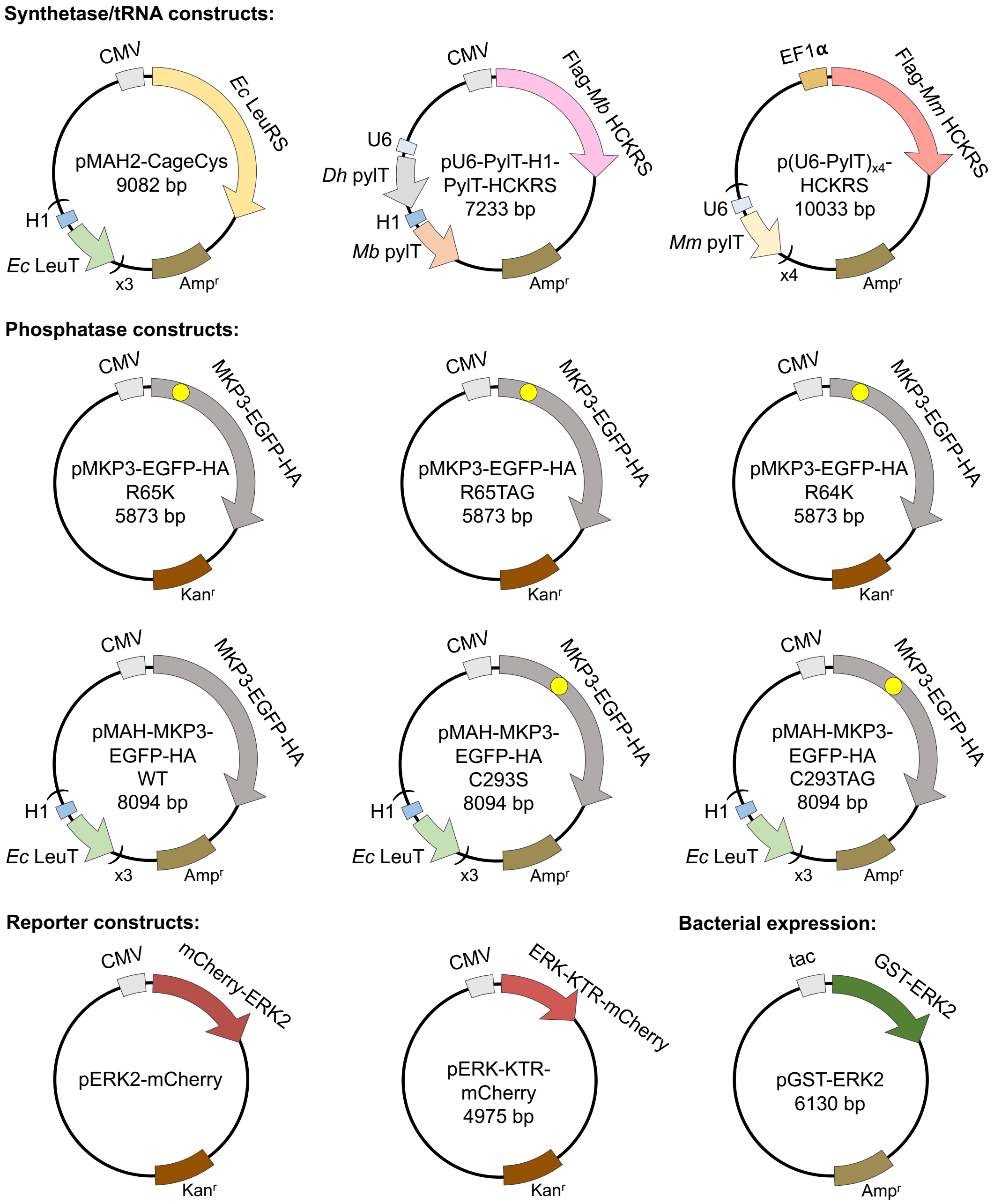

Supplementary Figure 3. Plasmid maps of all constructs used in this study. Plasmids pMAH2-CageCys and pERK2-mCherry were gifts from Huiwang Ai and Jason Haugh, respectively. The remaining plasmids were assembled as described in the Methods section.

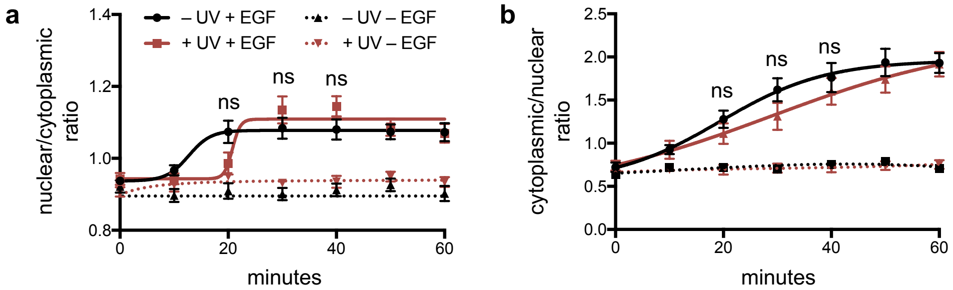

Supplementary Figure 4. To validate that photo-activation did not adversely affect cell health or, more importantly, interfere with the MAPK/ERK pathway, control experiments combining UV irradiation with or without EGF stimulation were performed for both reporters. a) The nuclear/cytoplasmic ratio over time for the ERK-mCherry reporter in the presence or absence of UV irradiation with or without EGF treatment (100 ng/mL) shows no significance difference between the irradiated and non-irradiated samples. b) The cytoplasmic/nuclear ratio over time for the ERK-KTR-mCherry reporter in the presence or absence of UV irradiation with or without EGF treatment (100 ng/mL) shows no significant difference between the irradiated and non-irradiated samples. Error bars represent standard error of the mean for nine individual cells obtained from three biological replicates. One-way ANOVA was used to compare the +/–UV samples; $p > 0.05$ equals ns. Data are provided in the Source Data file.

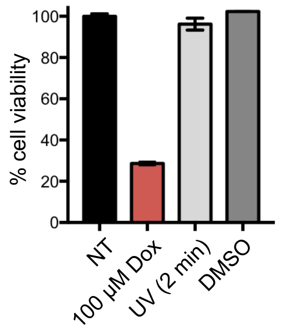

Supplementary Figure 5. Cell viability of HEK293T cells was performed to ensure that the UV irradiation did not adversely affect cell health. HEK293T cells were left non-treated (NT), treated with 100 μM doxorubicin (as a positive control for toxicity), irradiated for 2 minutes with UV light, or treated with 0.1% DMSO, then incubated for 72 hours. An XTT cell viability test was performed and values were normalized to the non-treated sample. Error bars represent standard deviation of experiment conducted in triplicate. No effect on cell viability was observed with the UV irradiation. Data are provided in the Source Data file.

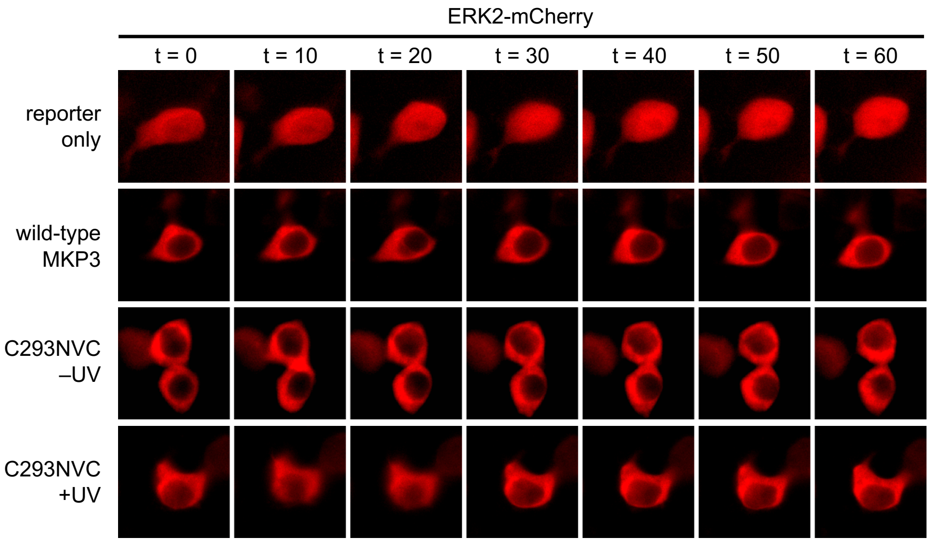

Supplementary Figure 6. The full 0 to 60-minute time course micrographs corresponding to the reporter images in Figure 2c.

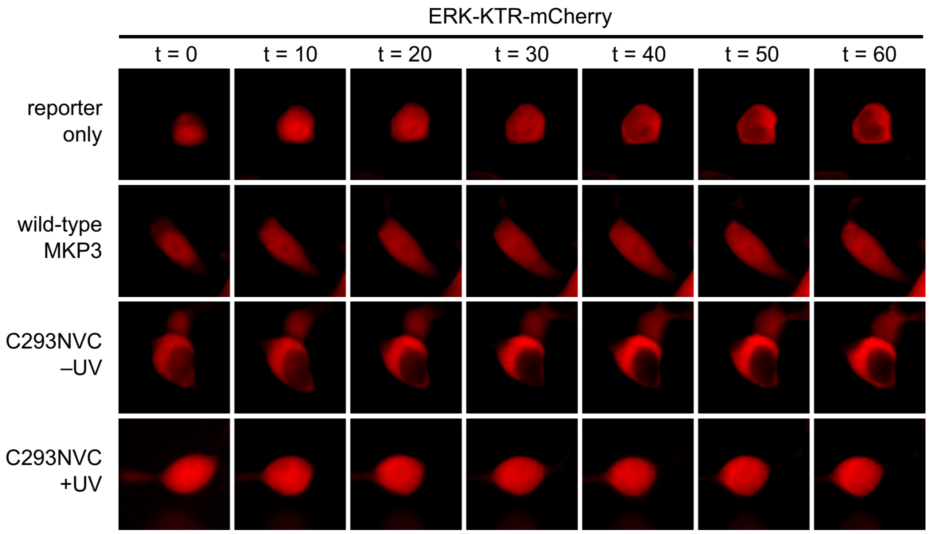

Supplementary Figure 7. The full 0 to 60-minute time course micrographs corresponding to the reporter images in Figure 3b.

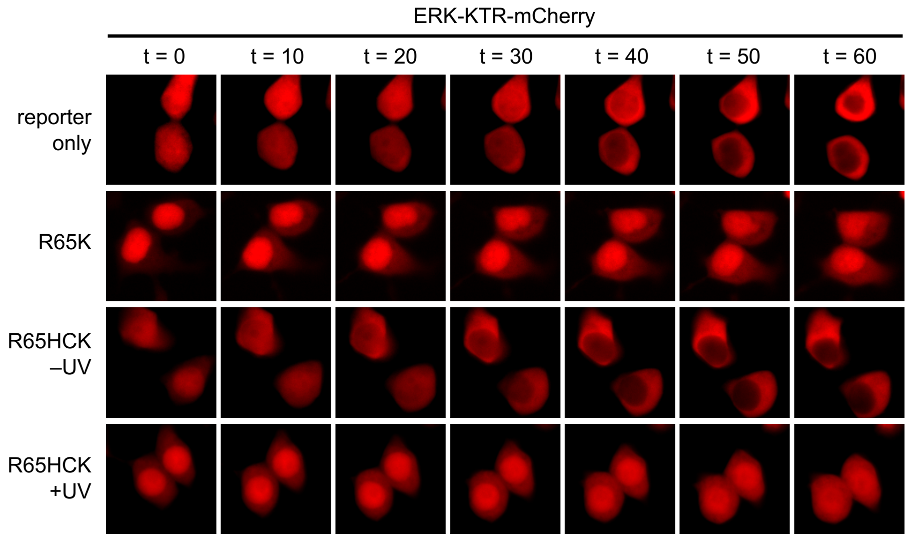

Supplementary Figure 8. The full 0 to 60-minute time course micrographs corresponding to the reporter images in Figure 5b.

Supplementary Table 1. List of primers used to generate DNA constructs. Restriction sites are indicated in bold, nucleotide mutations are indicated with capital letters, and the HA tag DNA sequence is highlighted in gray.

Primer	Sequence (5' → 3')
P1	aga**aagctt**atgatagatacgctcagacccgtgccc
P2	tct**ggatcc**cccgtagattgcagagagtccacc
P3	gcatggacgagctgtacaagtacccatacgatgttccagattacgcttaaagcggccgcgactctagatcataatcagc
P4	cttgtacagctcgtccatgccgagagtgatcccggcggcggtc
P5	gcat**gagctc**tcaagcttatgatagatacg
P6	cgta**gtttaaac**ttaagcgtaatctggaacat
P7	caagaactgtggtgtcttggtacatTAGttggctggc
P8	gcggctaatgccagccaaCTAatgtacc
P9	gtacatAGCttggctggcattagccgctca
P10	agccaaGCTatgtaccaagacaccacagttcttg
P11	ctgcggAAGctgcagaagggtaacctgccggtgc
P12	ctgcagCTTccgcagcatgatgcccgggatggc
P13	ctgcggTAGctgcagaagggtaacctgccggtgc
P14	ctgcagCTAccgcagcatgatgcccgggatggc
P15	atgctgAAGcgcctgcagaagggtaacctgccggt
P16	caggcgCTTcagcatgatgcccgggatggccac
P17	gatccaccggtcgccaccatgaagggccgaaagcct
P18	ctcgcccttgctcaccatactagtggatgggaattg
P19	caattcccatccactagtatggtgagcaagggcgag
P20	aggctttcggcccttcatggtggcgaccggtggatc

Supplementary References:

1 Regot, S., Hughey, J. J., Bajar, B. T., Carrasco, S. & Covert, M. W. High-sensitivity measurements of multiple kinase activities in live single cells. *Cell* **157**, 1724-1734, (2014).
