# Supplementary material for: Optical control of protein phosphatase function
Source: Nat Commun. 2019 Sep 26;10:4384. doi: 10.1038/s41467-019-12260-z (PMC6763421; doi:10.1038/s41467-019-12260-z)
Supplement: Supplementary file 2 — Reporting Summary [file 41467_2019_12260_MOESM2_ESM.pdf]

nature research

Corresponding author(s): Alexander Deiters

Last updated by author(s): Aug 6, 2019

Reporting Summary

Nature Research wishes to improve the reproducibility of the work that we publish. This form provides structure for consistency and transparency in reporting. For further information on Nature Research policies, see Authors & Referees and the Editorial Policy Checklist.

Statistics

For all statistical analyses, confirm that the following items are present in the figure legend, table legend, main text, or Methods section.

n/a	Confirmed	
☐	☒	The exact sample size (n) for each experimental group/condition, given as a discrete number and unit of measurement
☐	☒	A statement on whether measurements were taken from distinct samples or whether the same sample was measured repeatedly
☐	☒	The statistical test(s) used AND whether they are one- or two-sided<br>*Only common tests should be described solely by name; describe more complex techniques in the Methods section.*
☒	☐	A description of all covariates tested
☒	☐	A description of any assumptions or corrections, such as tests of normality and adjustment for multiple comparisons
☐	☒	A full description of the statistical parameters including central tendency (e.g. means) or other basic estimates (e.g. regression coefficient) AND variation (e.g. standard deviation) or associated estimates of uncertainty (e.g. confidence intervals)
☒	☐	For null hypothesis testing, the test statistic (e.g. F, t, r) with confidence intervals, effect sizes, degrees of freedom and P value noted<br>*Give P values as exact values whenever suitable.*
☒	☐	For Bayesian analysis, information on the choice of priors and Markov chain Monte Carlo settings
☒	☐	For hierarchical and complex designs, identification of the appropriate level for tests and full reporting of outcomes
☒	☐	Estimates of effect sizes (e.g. Cohen's d, Pearson's r), indicating how they were calculated

Our web collection on statistics for biologists contains articles on many of the points above.

Software and code

Policy information about availability of computer code

Data collection	Zen 2 (blue edition) was used for image acquisition. Tecan i-control was used for plate reader measurements. Image Lab (version 5.0) was used for Western blot images.
Data analysis	FIJI (ImageJ, version 2.0) was used for image analysis and export. Image Lab (version 5.0) was used for exporting Western blot images. Microsoft Excel (16.16.2) and Prism 7.0 were used for mathematical analysis and graphing of microscopy data.

For manuscripts utilizing custom algorithms or software that are central to the research but not yet described in published literature, software must be made available to editors/reviewers. We strongly encourage code deposition in a community repository (e.g. GitHub). See the Nature Research guidelines for submitting code & software for further information.

Data

Policy information about availability of data

All manuscripts must include a data availability statement. This statement should provide the following information, where applicable:

- Accession codes, unique identifiers, or web links for publicly available datasets
- A list of figures that have associated raw data
- A description of any restrictions on data availability

The data that support the findings of this study are available from the authors upon reasonable request.

Field-specific reporting

Please select the one below that is the best fit for your research. If you are not sure, read the appropriate sections before making your selection.

☒ Life sciences ☐ Behavioural & social sciences ☐ Ecological, evolutionary & environmental sciences

Life sciences study design

All studies must disclose on these points even when the disclosure is negative.

Sample size	For biochemical assays, three biological (independent) replicates were used. For bulk population analyses, sample size of 40-45 was used for caged samples and sample size of 85-90 was used for control samples.
Data exclusions	No data were excluded from the analyses.
Replication	Microscopy experiments were performed over the course of several months and were successfully reproduced.
Randomization	No randomization was required for the experiments performed within.
Blinding	No blinding was required.

Reporting for specific materials, systems and methods

We require information from authors about some types of materials, experimental systems and methods used in many studies. Here, indicate whether each material, system or method listed is relevant to your study. If you are not sure if a list item applies to your research, read the appropriate section before selecting a response.

Materials & experimental systems

n/a	Involved in the study	
☐	☒	Antibodies
☐	☒	Eukaryotic cell lines
☒	☐	Palaeontology
☒	☐	Animals and other organisms
☒	☐	Human research participants
☒	☐	Clinical data

Methods

n/a	Involved in the study	
☒	☐	ChIP-seq
☒	☐	Flow cytometry
☒	☐	MRI-based neuroimaging

Antibodies

Antibodies used	anti-GFP (ProteinTech 50430-2-AP, rabbit polyclonal)<br>anti-GAPDH (ProteinTech 10494-1-AP, rabbit polyclonal)<br>goat anti-rabbit HRP-conjugate (ProteinTech SA00001-2)
Validation	Both primary antibodies were validated by the manufacturer and have been previously cited in the literature.

Eukaryotic cell lines

Policy information about cell lines

Cell line source(s)	HEK293T (ATCC, cat. no. CRL-11268)
Authentication	None of the cell lines used were authenticated.
Mycoplasma contamination	HEK293T cells were tested every 3 months for mycoplasma contamination (Genlantis MycoScope PCR Kit). Cells tested negative each time.
Commonly misidentified lines (See ICLAC register)	No commonly misidentified cell lines were used.
